# Optimal glucose, HbA1c, glucose-HbA1c ratio and stress-hyperglycaemia ratio cut-off values for predicting 1-year mortality in diabetic and non-diabetic acute myocardial infarction patients

**DOI:** 10.1186/s12933-021-01395-3

**Published:** 2021-10-19

**Authors:** Ching-Hui Sia, Mervyn Huan-Hao Chan, Huili Zheng, Junsuk Ko, Andrew Fu-Wah Ho, Jun Chong, David Foo, Ling-Li Foo, Patrick Zhan-Yun Lim, Boon Wah Liew, Ping Chai, Tiong-Cheng Yeo, Huay-Cheem Tan, Terrance Chua, Mark Yan-Yee Chan, Jack Wei Chieh Tan, Heerajnarain Bulluck, Derek J. Hausenloy

**Affiliations:** 1 Department of Cardiology, National University Heart Centre Singapore, Singapore, Singapore; 2 Yong Loo Lin School of Medicine, National University of Singapore, Singapore, Singapore; 3 Cardiovascular & Metabolic Disorders Program, Duke-NUS Medical School, 8 College Road, Level 8, Singapore, 169857 Singapore; 4 Health Promotion Board, National Registry of Diseases Office, Singapore, Singapore; 5 MD Program, Duke-NUS Medical School, Singapore, Singapore; 6 SingHealth Duke-NUS Emergency Medicine Academic Clinical Programme, Singapore, Singapore; 7 National Heart Research Institute Singapore, National Heart Centre Singapore, Singapore, Singapore; 8 Pre-Hospital and Emergency Care Research Centre, Health Services and Systems Research, Duke-NUS Medical School, Singapore, Singapore; 9 Department of Cardiology, National Heart Centre Singapore, Singapore, Singapore; 10 Tan Tock Seng Hospital, Singapore, Singapore; 11 Khoo Teck Puat Hospital, Singapore, Singapore; 12 Changi General Hospital, Singapore, Singapore; 13 Leeds Institute of Cardiovascular and Metabolic Medicine, University of Leeds, Leeds, UK; 14 Department of Cardiology, Leeds General Infirmary, Leeds Teaching Hospitals NHS Trust, Leeds, UK; 15 The Hatter Cardiovascular Institute, University College London, London, UK; 16 Cardiovascular Research Center, College of Medical and Health Sciences, Asia University, Taichung City, Taiwan

## Abstract

**Background:**

Stress-induced hyperglycaemia at time of hospital admission has been linked to worse prognosis following acute myocardial infarction (AMI). In addition to glucose, other glucose-related indices, such as HbA1c, glucose-HbA1c ratio (GHR), and stress-hyperglycaemia ratio (SHR) are potential predictors of clinical outcomes following AMI. However, the optimal blood glucose, HbA1c, GHR, and SHR cut-off values for predicting adverse outcomes post-AMI are unknown. As such, we determined the optimal blood glucose, HbA1c, GHR, and SHR cut-off values for predicting 1-year all cause mortality in diabetic and non-diabetic ST-segment elevation myocardial infarction (STEMI) and non-ST-segment elevation myocardial infarction (NSTEMI) patients.

**Methods:**

We undertook a national, registry-based study of patients with AMI from January 2008 to December 2015. We determined the optimal blood glucose, HbA1c, GHR, and SHR cut-off values using the Youden’s formula for 1-year all-cause mortality. We subsequently analyzed the sensitivity, specificity, positive and negative predictive values of the cut-off values in the diabetic and non-diabetic subgroups, stratified by the type of AMI.

**Results:**

There were 5841 STEMI and 4105 NSTEMI in the study. In STEMI patients, glucose, GHR, and SHR were independent predictors of 1-year all-cause mortality [glucose: OR 2.19 (95% CI 1.74–2.76); GHR: OR 2.28 (95% CI 1.80–2.89); SHR: OR 2.20 (95% CI 1.73–2.79)]. However, in NSTEMI patients, glucose and HbA1c were independently associated with 1-year all-cause mortality [glucose: OR 1.38 (95% CI 1.01–1.90); HbA1c: OR 2.11 (95% CI 1.15–3.88)]. In diabetic STEMI patients, SHR performed the best in terms of area-under-the-curve (AUC) analysis (glucose: AUC 63.3%, 95% CI 59.5–67.2; GHR 68.8% 95% CI 64.8–72.8; SHR: AUC 69.3%, 95% CI 65.4–73.2). However, in non-diabetic STEMI patients, glucose, GHR, and SHR performed equally well (glucose: AUC 72.0%, 95% CI 67.7–76.3; GHR 71.9% 95% CI 67.7–76.2; SHR: AUC 71.7%, 95% CI 67.4–76.0). In NSTEMI patients, glucose performed better than HbA1c for both diabetic and non-diabetic patients in AUC analysis (For diabetic, glucose: AUC 52.8%, 95% CI 48.1–57.6; HbA1c: AUC 42.5%, 95% CI 37.6–47. For non-diabetic, glucose: AUC 62.0%, 95% CI 54.1–70.0; HbA1c: AUC 51.1%, 95% CI 43.3–58.9). The optimal cut-off values for glucose, GHR, and SHR in STEMI patients were 15.0 mmol/L, 2.11, and 1.68 for diabetic and 10.6 mmol/L, 1.72, and 1.51 for non-diabetic patients respectively. For NSTEMI patients, the optimal glucose values were 10.7 mmol/L for diabetic and 8.1 mmol/L for non-diabetic patients.

**Conclusions:**

SHR was the most consistent independent predictor of 1-year all-cause mortality in both diabetic and non-diabetic STEMI, whereas glucose was the best predictor in NSTEMI patients.

**Supplementary Information:**

The online version contains supplementary material available at 10.1186/s12933-021-01395-3.

## Introduction

Stress-induced hyperglycaemia (SH) refers to the transient rise in blood glucose levels that occurs during an acute illness and has been linked to a worse prognosis in acute myocardial infarction (AMI) patients [1]. Despite its potential role as a predictor for patient outcomes, guidelines are not consistent on the choice of optimal glucose level to define SH as the thresholds have been arbitrarily selected due to lack of scientific evidence. As such, the prognostic relevance of the blood glucose levels for defining SH are not clear and need to be better defined. The European Society of Cardiology and the American Heart Association recommend an admission blood glucose of > 11 mmol/L and > 10 mmol/L as cut-off values for defining SH, respectively, regardless of diabetic or chronic glycaemic status of patients [2, 3]. Previous therapeutic trials for improving acute glucose control in AMI patients have been inconsistent in their definitions of glucose level that constitutes SH, which might account, in part, for the inconclusive results of these studies in terms of clinical outcomes [4–6].

This uncertainty in optimal cut-off values for glucose in AMI patients in predicting adverse events, may also differ between ST-segment elevation myocardial infarction (STEMI) and non-ST-segment elevation myocardial infarction (NSTEMI) patients, and there is also a need to account for the diabetic status of patients to avoid incorrect estimation of real prevalence of stress hyperglycaemia. Roberts et al. [7] have devised a Stress Hyperglycaemia Ratio (SHR) index to normalise the acute increase in glucose values in relation to background glycaemic status, but the optimal SHR cut-off level for defining SH are not known. Similarly, hemoglobin a1c (HbA1c) and glucose-HbA1c-ratio (GHR) were reported as predictors of clinical outcomes in AMI and other pathological processes, but their relative performance to other predictors and optimal values were not clarified [8, 9].

As such, in this study, we evaluated and compared the optimal blood glucose and SHR cut-off values in both diabetic and non-diabetic STEMI and NSTEMI patients and their utility in predicting 1-year all-cause mortality as compared to other potential predictors, such as glucose, HbA1c, and GHR.

## Methods

This study utilised the Singapore Myocardial Infarction Registry (SMIR), a national registry managed by the ministry-funded National Registry of Diseases Office. The local institutional review board granted an exemption for written consent from the participants for this study (SingHealth CIRB Reference No: 2016/2480) as this study utilised de-identified data. The research was conducted in accordance with the Declaration of Helsinki. The statistician had access to anonymised individual data, while the co-authors had access to analysed, aggregated data. The SMIR collects clinical data of all AMI patients in all hospitals in Singapore [10–14]. Notification of AMI to the registry is mandated by law. The International Classification of Diseases, 9th Revision, Clinical Modification (ICD-9-CM) code 410 was used to obtain AMI cases diagnosed prior to 2012, while ICD-10 (Australian Modification) codes I21 and I22 were used for those cases diagnosed in 2012. Patients’ data was extracted from medical claims listings, hospital discharge summaries, and medical records by dedicated registry coordinators. Annual audit was performed on the SMIR data for accuracy and inter-rater reliability, with outliers and illogical data flagged for review. The multinational monitoring of trends and determinants in cardiovascular disease criteria were used to define episodes. STEMI was defined by: (1) Typical chest pain of 20 min, (2) Significant ST segment elevation (0.1 or 0.2 mV on 2 adjacent limb or precordial leads, respectively, or new left bundle-branch block) and (3) Confirmed later by a rise in biomarkers. Medication use was based on documentation in the medical records. The SMIR data was subsequently merged with data from the national death registry, which captures all deaths in Singapore to obtain the outcome of interest—1-year all-cause mortality.

**Fig. 1:**
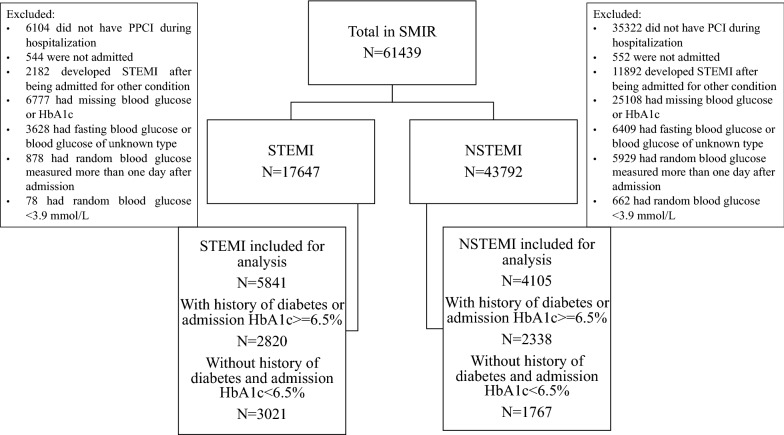
Flow chart of inclusion and exclusion criteria. NSTEMI, non-ST-segment elevation myocardial infarction; PPCI, primary percutaneous coronary intervention; SMIR, Singapore Myocardial Infarction Registry; STEMI, ST-segment elevation myocardial infarction

This study utilised STEMI and NSTEMI cases reported to the SMIR from January 2008 to December 2015 who received percutaneous coronary intervention [15]. We excluded patients with a blood glucose level of < 3.9 mmol/L, a first glucose level measured more than 24 h after admission, patients with fasting glucose levels, patients managed outside the hospital and patients with missing glucose or HbA1c results (Fig. 1). The glucose values referred henceforth throughout the manuscript implies the admission random glucose within the first 24 h. Diabetics were defined as patients with a previously documented history of diabetes or those with no documented history of diabetes but a HbA1c value of > 6.5% [16]. Non-diabetics were defined as those without a history of diabetes and with a HbA1c value of ≤ 6.5%.

### Statistical analysis

Categorical variables of the patients’ characteristics were expressed as frequency and percentages while continuous variables were expressed as median and interquartile range. The SHR, was calculated using the following formula:

$$SHR = \frac{{Acute\;glucose\;value \left( {mmol/L} \right)}}{{\left[ {1.59 \times HbA1c \left( \% \right)} \right] - 2.59}}$$

The GHR, the ratio of glucose to HbA1c, was calculated as performed in the previous report [17].

The optimal cut-off value for each glucose, HbA1c, and GHR and SHR metric were determined by the Youden’s index [18]. A 2 × 2 table was used to determine the sensitivity, specificity, positive and negative predictive value of the cut-off values. As mortality varied with time, time-dependent receiver operating characteristic (ROC) curves with inverse probability of censoring weighting were generated to compare the area-under-the-curves (AUC) of each metric [19–22]. Missing data were excluded from the analyses through case deletion without imputation to maintain data in its original form. To determine if glucose and SHR were independent predictors of 1-year all-cause mortality, odd ratios (OR) with 95% confidence interval (95% CI) were adjusted for age, a history of ischemic heart disease, Killip class on admission, cardiac arrest on admission, creatinine on admission and hemoglobin on admission (factors found to be significant predictors of 1-year all-cause mortality using multivariable stepwise logistic regression with backward elimination) [10–14]. Statistical analyses were performed using Stata SE version 13 and R verison 4.0.3, with statistical significance was set at p < 0.05.

## Results

### Baseline characteristics

A total of 5841 STEMI and 4105 NSTEMI patients were included in the analysis (Fig. 1). Patients were divided into the diabetic and non-diabetic subgroups. Baseline patient characteristics are displayed in Table 1. Diabetic patients were older, less likely to be male. There were more patients with a past medical history of hypertension, hyperlipidaemia, and ischaemic heart disease in the diabetic group, but fewer were current smokers. Median glucose levels were higher in STEMI patients. The proportion of patients on goal-directed medical therapy was high for both the STEMI and NSTEMI groups.

**Table 1:** Characteristics of acute myocardial infarction patients in the study

	STEMI		NSTEMI	
	Diabetic N = 2820	Non-diabetic N = 3021	Diabetic N = 2338	Non-diabetic N = 1767
Age in years, median (IQR)	58.6 (51.4–66.2)	57.0 (50.5–64.9)	61.2 (54.0–70.0)	58.4 (50.9–66.9)
Male, n (%)	2289 (81.2)	2644 (87.5)	1695 (72.5)	1498 (84.8)
Race, n (%)				
Chinese	1457 (51.7)	1997 (66.1)	1208 (51.7)	1202 (68.0)
Malay	714 (25.3)	574 (19.0)	550 (23.5)	305 (17.3)
Indian	601 (21.3)	400 (13.2)	540 (23.1)	230 (13.0)
Past medical history, n (%)				
Hypertension	1854 (65.7)	1266 (41.9)	1876 (80.2)	963 (54.5)
Diabetes	2142 (76.0)	NA	1994 (85.3)	NA
Not on treatment	451 (21.1)	NA	257 (12.9)	NA
Diet control	113 (5.3)	NA	102 (5.1)	NA
Oral medication	1391 (64.9)	NA	1264 (63.4)	NA
Insulin	64 (3.0)	NA	153 (7.7)	NA
Oral medication & insulin	123 (5.7)	NA	218 (10.9)	NA
Hyperlipidemia	1701 (60.4)	1070 (35.4)	1825 (78.1)	906 (51.3)
MI/PCI/CABG	499 (17.7)	319 (10.6)	963 (41.2)	337 (19.1)
Smoking, n (%)				
Current	1253 (44.9)	1602 (53.3)	750 (32.2)	806 (45.7)
Former	422 (15.1)	365 (12.1)	468 (20.1)	315 (17.9)
Never	1118 (40.0)	1039 (34.6)	1113 (47.7)	641 (36.4)
BMI in kg/m^2^, median (IQR)	25.2 (22.9–27.9)	24.4 (22.2–27.0)	25.6 (23.1–28.9)	24.8 (22.6–27.5)
Glucose in mmol/L, median (IQR)	13.8 (10.5–18.4)	7.8 (6.8–9.4)	12.3 (9.0–16.4)	6.7 (5.8–8.0)
HbA1c in %, median (IQR)	8.0 (6.9–9.9)	5.7 (5.5–6.0)	7.7 (6.8–9.3)	5.7 (5.5–6.0)
Total cholesterol in mmol/L, median (IQR)	4.9 (4.0–5.9)	5.2 (4.5–6.1)	4.7 (3.8–5.7)	5.2 (4.4–6.0)
LDL-cholesterol in mmol/L, median (IQR)	3.1 (2.4–4.0)	3.5 (2.8–4.3)	2.8 (2.2–3.7)	3.5 (2.7–4.2)
HDL-cholesterol in mmol/L, median (IQR)	1.0 (0.8–1.2)	1.1 (0.9–1.3)	1.0 (0.8–1.2)	1.0 (0.9–1.2)
Triglyceride in mmol/L, median (IQR)	1.5 (1.1–2.3)	1.3 (1.0–1.8)	1.7 (1.2–2.5)	1.5 (1.1–2.1)
Haemoglobin in g/dL, median (IQR)	14.6 (13.2–15.7)	14.8 (13.7–15.8)	13.7 (12.1–15.0)	14.5 (13.4–15.4)
Creatinine in µmol/L, median (IQR)	89 (74–112)	90 (77–105)	88 (72–116)	84 (73–97)
Killip class on admission, n (%)				
I	2261 (80.2)	2541 (84.1)	1790 (76.6)	1599 (90.5)
II	196 (6.9)	126 (4.2)	322 (13.8)	94 (5.3)
III	130 (4.6)	86 (2.9)	199 (8.5)	52 (2.9)
IV	233 (8.3)	267 (8.8)	26 (1.1)	21 (1.2)
In-patient medication, n (%)				
Aspirin	2733 (96.9)	2956 (97.9)	2298 (98.3)	1722 (97.5)
Beta-blocker	2457 (87.1)	2616 (86.6)	2121 (90.7)	1575 (89.1)
ACEI/ARB	2171 (77.0)	2221 (73.5)	1930 (82.6)	1265 (71.6)
Lipid lowering drug	2731 (96.8)	2955 (97.8)	2313 (98.9)	1755 (99.3)
P2Y12 inhibitor	2773 (98.3)	2982 (98.7)	2330 (99.7)	1760 (99.6)

*ACEI/ARB* angiotensin converting enzyme inhibitor/angiotensin receptor blocker, *BMI* body mass index, *CABG* coronary artery bypass grafting, *IQR* interquartile range, *MI* myocardial infarction, *NA* not applicable, *NSTEMI* non-ST-segment elevation myocardial infarction, *PCI* percutaneous coronary intervention, *SHR* stress-hyperglycaemia ratio, *STEMI* ST-segment elevation myocardial infarction

### General trend of survival, hazard ratio, and mortality in STEMI and NSTEMI patients with or without diabetes

One-year all-cause mortality occurred in: 252 out of 2820 (8.9%) diabetic STEMI patients; 202 out of 3021 (6.7%) non-diabetic STEMI patients; 161 out of 2338 (6.9%) diabetic NSTEMI patients; 56 out of 1767 (3.2%) non-diabetic NSTEMI patients. The survival rate rapidly dropped within the first 30 days after AMI events in STEMI and NSTEMI patients with STEMI being worse (Additional file 1: Figure S1A) as previously reported by other researchers [19]. Non-diabetic patients in STEMI and NSTEMI groups had significantly better survival rates than diabetic patients (Additional file 1: Figure S1B). Generally, there was an exponential relationship between glucose and hazard ratio (HR) in all sub-groups of STEMI and NSTEMI with non-diabetic STEMI being associated with the greatest HR (Additional file 1: Figure S2). Similarly, SHR was associated with increased HR exponentially in all sub-groups of STEMI and NSTEMI (Additional file 1: Figure S3). Glucose was correlated with 1-year all-cause mortality in non-diabetic AMI patients in a concentration-dependent manner (Additional file 1: Figure S4a). Similarly, elevated levels of GHR were associated with increased 1-year all-cause mortality in both diabetic and non-diabetic AMI patients (Additional file 1: Figure S4c). A HbA1c of < 5.0% was correlated with increased 1-year all-cause mortality in diabetic AMI. However, this concentration-dependent association was not found for HbA1c (Additional file 1: Figure S4b-i and b-ii).

### Optimal blood glucose cut-off values for predicting outcomes

The optimal glucose cut-off values for predicting all-cause mortality at 1 year are shown in Table 2. All cut-off values for both STEMI and NSTEMI patients, regardless of diabetic status showed excellent negative predictive value of > 94%. The optimal glucose cut-off values were 15.0 mmol/L for diabetic STEMI patients and 10.6 mmol/L for non-diabetic STEMI patients. In NSTEMI patients, the optimal cut-off values were lower, at 10.7 mmol/L for diabetic patients and 8.1 mmol/L for non-diabetic patients.

**Table 2:** Optimal glucose cut-off values and performance of the cut-off values in predicting 1-year all-cause mortality in diabetic and non-diabetic ST-segment elevation myocardial infarction and non-ST-segment elevation myocardial infarction patients

	STEMI		NSTEMI	
	Diabetic	Non-diabetic	Diabetic	Non-diabetic
Statistical optimal glucose cut-off in mmol/L	15.0	10.6	10.7	8.1
Sensitivity in % (95% CI)	62.7 (56.4–68.7)	50.5 (43.4–57.6)	65.8 (58.0–73.1)	42.9 (29.7–56.8)
Specificity in % (95% CI)	59.5 (57.5–61.4)	86.9 (85.6–88.1)	39.7 (37.6–41.8)	75.8 (73.7–77.8)
Positive predictive value in % (95% CI)	13.2 (12.0–14.4)	21.6 (18.9–24.6)	7.5 (6.7–8.3)	5.5 (4.1–7.4)
Negative predictive value in % (95% CI)	94.2 (93.2–95.0)	96.1 (95.5–96.6)	94.0 (92.7–95.1)	97.6 (97.0–98.1)

*CI* confidence interval, *NSTEMI* non-ST-segment elevation myocardial infarction, *STEMI* ST-segment elevation myocardial infarction

### Optimal HbA1c cut-off values for predicting outcomes

The optimal HbA1c cut-off values for predicting all-cause mortality at 1 year are shown in Table 3. All cut-off values for both STEMI and NSTEMI patients, regardless of diabetic status showed excellent negative predictive value of > 91%. The optimal HbA1c cut-off values were 13.3 mmol/L for diabetic STEMI patients and 6.0 mmol/L for non-diabetic STEMI patients. In NSTEMI patients, the optimal cut-off values were 12.2 mmol/L for diabetic patients and 6.3 mmol/L for non-diabetic patients.

**Table 3:** Optimal HbA1c cut-off values and performance of the cut-off values in predicting 1-year all-cause mortality in diabetic and non-diabetic ST-segment elevation myocardial infarction and non-ST-segment elevation myocardial infarction patients

	STEMI		NSTEMI	
	Diabetic	Non-diabetic	Diabetic	Non-diabetic
Statistical optimal HbA1c cut-off in mmol/L	13.3	6.0	12.2	6.3
Sensitivity in % (95% CI)	4.0 (1.6–6.4)	27.7 (21.6–33.9)	4.3 (1.2–7.5)	14.3 (5.1–23.5)
Specificity in % (95% CI)	97.6 (97.0–98.2)	75.1 (73.5–76.7)	96.5 (95.7–97.2)	92.2 (91.0–93.5)
Positive predictive value in % (95% CI)	14.1 (6.0–22.2)	7.4 (5.5–9.3)	8.3 (2.4–14.2)	5.7 (1.9–9.5)
Negative predictive value in % (95% CI)	91.2 (90.1–92.3)	93.6 (92.5–94.6)	93.2 (92.1–94.2)	97.0 (96.2–97.9)

*CI* confidence interval, *NSTEMI* non-ST-segment elevation myocardial infarction, *STEMI* ST-segment elevation myocardial infarction

### Optimal glucose-HbA1c ratio (GHR) cut-off values for predicting outcomes

The optimal GHR cut-off values for predicting all-cause mortality at 1 year are shown in Table 4. All cut-off values for both STEMI and NSTEMI patients, regardless of diabetic status showed excellent negative predictive value of > 94%. The optimal GHR cut-off values were 2.11 mmol/L for diabetic STEMI patients and 1.72 mmol/L for non-diabetic STEMI patients. In NSTEMI patients, the optimal cut-off values were lower, at 2.10 mmol/L for diabetic patients and 1.43 mmol/L for non-diabetic patients.

**Table 4:** Optimal glucose-HbA1c ratio (GHR) cut-off values and performance of the cut-off values in predicting 1-year all-cause mortality in diabetic and non-diabetic ST-segment elevation myocardial infarction and non-ST-segment elevation myocardial infarction patients

	STEMI		NSTEMI	
	Diabetic	Non-diabetic	Diabetic	Non-diabetic
Statistical optimal GHR cut-off in mmol/L	2.11	1.72	2.10	1.43
Sensitivity in % (95% CI)	50.4 (44.2–56.6)	55.0 (48.1–61.8)	28.6 (21.6–35.6)	41.1 (28.2–54.0)
Specificity in % (95% CI)	82.5 (81.0–83.9)	82.7 (81.3–84.1)	84.1 (82.5–85.6)	78.1 (76.1–80.0)
Positive predictive value in % (95% CI)	22.0 (18.6–25.4)	18.5 (15.4–21.6)	11.7 (8.5–14.9)	5.8 (3.5–8.1)
Negative predictive value in % (95% CI)	94.4 (93.5–95.4)	96.2 (95.5–97.0)	94.1 (93.0–95.1)	97.6 (96.8–98.4)

*CI* confidence interval, *GHR* glucose-hb1ac ratio, *NSTEMI* non-ST-segment elevation myocardial infarction, *STEMI* ST-segment elevation myocardial infarction

### Optimal SHR cut-off values for predicting outcomes

The optimal SHR cut-off values for predicting all-cause mortality at 1 year are shown in Table 5. The optimal cut-off values performed well with negative predictive values of > 94%. The SHR cut-off value for diabetic STEMI patients was 1.68, and was lower in non-diabetic STEMI patients at 1.51. For the NSTEMI group, the SHR values were lower being 1.53 in diabetics and 1.27 in non-diabetics.

**Table 5:** Optimal stress-hyperglycaemia ratio (SHR) cut-off values and performance of the cut-off values in predicting 1-year all-cause mortality in diabetic and non-diabetic ST-segment elevation myocardial infarction and non-ST-segment elevation myocardial infarction patients

	STEMI		NSTEMI	
	Diabetic	Non-diabetic	Diabetic	Non-diabetic
Statistical optimal SHR cut-off in mmol/L	1.68	1.51	1.53	1.27
Sensitivity in % (95% CI)	50.4 (44.1–56.7)	55.0 (47.8–61.9)	35.4 (28.0–43.3)	44.6 (31.3–58.5)
Specificity in % (95% CI)	83.2 (81.7–84.6)	82.2 (80.7–83.6)	78.3 (76.5–80.0)	79.0 (77.0–80.9)
Positive predictive value in % (95% CI)	22.7 (20.2–25.5)	18.1 (16.0–20.4)	10.8 (8.8–13.1)	6.5 (4.9–8.6)
Negative predictive value in % (95% CI)	94.5 (93.8–95.1)	96.2 (95.6–96.7)	94.2 (93.6–94.9)	97.8 (97.2–98.2)

*CI* confidence interval, *SHR* stress-hyperglycaemia ratio, *NSTEMI* non-ST-segment elevation myocardial infarction, *STEMI* ST-segment elevation myocardial infarction

### Comparison of optimal glucose, HbA1c, GHR, and SHR cut-off values

**Fig. 2:**
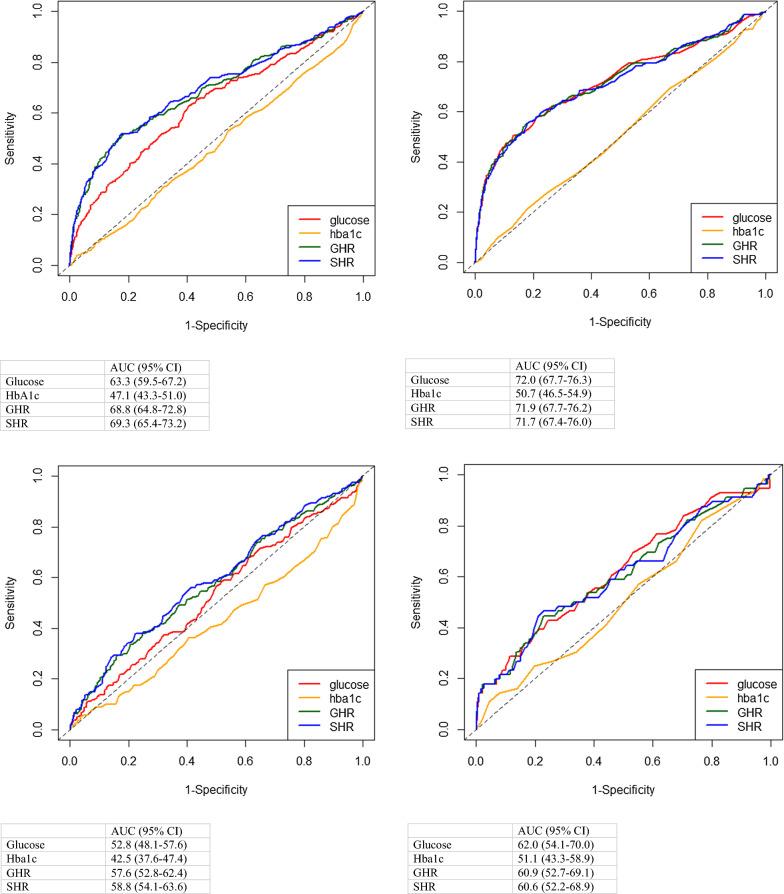
**a** Area under the curve for glucose, HbA1c, glucose-HbA1c ratio (GHR) and stress-hyperglycemia ratio (SHR) among diabetic STEMI patients. **b** Area under the curve for glucose, HbA1c, glucose-HbA1c ratio (GHR) and stress-hyperglycemia ratio (SHR) among non-diabetic STEMI patients. **c** Area under the curve for glucose, HbA1c, glucose-HbA1c ratio (GHR) and stress-hyperglycemia ratio (SHR) among diabetic NSTEMI patients. **d** Area under the curve for glucose, HbA1c, glucose-HbA1c ratio (GHR) and stress-hyperglycemia ratio (SHR) among non-diabetic NSTEMI patients

In STEMI patients, SHR was the consistent best predictor regardless of the diabetic condition (Fig. 2a, b). Glucose, GHR, and SHR performed equally well in diabetic patients (glucose: AUC 63.3%, 95% CI 59.5–67.2; HbA1c: AUC 47.1%, 95% CI 43.3–51.0; GHR 68.8% 95% CI 64.8–72.8; SHR: AUC 69.3%, 95% CI 65.4–73.2), whereas in non-diabetic patients, GHR and SHR performed equally well (glucose: AUC 72.0%, 95% CI 67.7–76.3; HbA1c: AUC 50.7%, 95% CI 46.5–54.9; GHR 71.9% 95% CI 67.7–76.2; SHR: AUC 71.7%, 95% CI 67.4–76.0).

In diabetic NSTEMI patients as shown in Fig. 2c, d, SHR was the best predictor of 1-year all-cause mortality (glucose: AUC 52.8%, 95% CI 48.1–57.46 HbA1c: AUC 42.5%, 95% CI 37.6–47.4; GHR 57.6% 95% CI 52.8–62.4; SHR: AUC 58.8%, 95% CI 54.1–63.6). However, in non-diabetic NSTEMI patients, glucose was the best predictor (glucose: AUC 62.0%, 95% CI 54.1–70.0 HbA1c: AUC 51.1%, 95% CI 43.3–58.9; GHR 60.9% 95% CI 52.7–69.1; SHR: AUC 60.6%, 95% CI 52.2–68.9).

### Glucose and SHR as an independent predictor of 1-year all-cause mortality

In STEMI patients, glucose, GHR, and SHR were independent predictors of 1-year all-cause mortality [glucose: OR 2.19 (95% CI 1.74–2.76); HbA1c: OR 1.00 (95% CI 0.73–1.36); GHR: OR 2.28 (95% CI 1.80–2.89); SHR: OR 2.20 (95% CI 1.73–2.79)], after adjusting for age, history of ischemic heart disease, Killip class on admission, cardiac arrest on admission, creatinine on admission and haemoglobin on admission (Tables 6, 7, 8, 9). However, in NSTEMI patients, glucose and HbA1c were independently associated with 1-year all-cause mortality [glucose: OR 1.38 (95% CI 1.01–1.90); HbA1c: OR 2.11 (95% CI 1.15–3.88); GHR: OR 1.30 (95% CI 0.92–1.83); SHR: OR 1.25 (95% CI 0.90–1.75)] (Tables 6, 7, 8, 9).

**Table 6:** Glucose as an independent predictor of 1-year all-cause mortality

	STEMI			NSTEMI		
	OR	95% CI		OR	95% CI	
Glucose						
Below proposed cut-off	1.00	Reference		1.00	Reference	
Above proposed cut-off	2.19	1.74	2.76	1.38	1.01	1.90
Age in years						
< 40	1.00	Reference		1.00	Reference	
40–49	1.25	0.47	3.32	0.32	0.07	1.45
50–59	1.46	0.57	3.77	0.42	0.11	1.64
60–69	2.29	0.89	5.90	0.72	0.19	2.75
70–79	4.17	1.60	10.88	1.20	0.32	4.55
≥ 80	8.46	3.16	22.69	2.10	0.54	8.25
History of MI/PCI/CABG						
No	1.00	Reference		1.00	Reference	
Yes	1.19	0.89	1.59	1.51	1.11	2.07
Killip class on admission						
I	1.00	Reference		1.00	Reference	
II	1.47	0.95	2.28	2.79	1.89	4.10
III	2.48	1.67	3.68	3.11	2.02	4.78
IV	4.15	3.15	5.46	21.34	10.67	42.71
CPR on arrival						
No	1.00	Reference		1.00	Reference	
Yes	3.89	2.74	5.53	10.48	3.34	32.95
Creatinine in µmol/L						
< 70	1.00	Reference		1.00	Reference	
70–105	0.80	0.55	1.15	0.71	0.43	1.17
106–140	1.75	1.20	2.56	1.36	0.80	2.32
141–176	2.28	1.43	3.66	1.20	0.61	2.35
177–353	2.74	1.66	4.52	1.21	0.58	2.55
≥ 354	3.89	2.07	7.34	4.47	2.47	8.07
Haemoglobin in g/dL						
< 10	1.00	Reference		1.00	Reference	
10–11	1.25	0.70	2.21	1.26	0.72	2.20
12–13	0.86	0.49	1.49	1.07	0.60	1.91
14–15	0.59	0.33	1.03	0.44	0.22	0.87
≥ 16	0.69	0.37	1.29	0.41	0.16	1.07

*CABG* coronary artery bypass grafting, *CI* confidence interval, *MI* myocardial infarction, *NSTEMI* non-ST-segment elevation myocardial infarction, *OR* odds ratio, *PCI* percutaneous coronary intervention, *STEMI* ST-segment elevation myocardial infarction

**Table 7:** HbA1c as an independent predictor of 1-year all-cause mortality

	STEMI			NSTEMI		
	OR	95% CI		OR	95% CI	
HbA1c						
Below proposed cut-off	1.00	Reference		1.00	Reference	
Above proposed cut-off	1.00	0.73	1.36	2.11	1.15	3.88
Age in years						
< 40	1.00	Reference		1.00	Reference	
40–49	1.32	0.50	3.53	0.35	0.08	1.59
50–59	1.52	0.59	3.93	0.45	0.12	1.76
60–69	2.35	0.91	6.06	0.79	0.21	3.04
70–79	4.19	1.60	10.96	1.28	0.33	4.90
≥ 80	8.60	3.20	23.10	2.21	0.56	8.78
History of MI/PCI/CABG						
No	1.00	Reference		1.00	Reference	
Yes	1.14	0.85	1.51	1.56	1.14	2.14
Killip class on admission						
I	1.00	Reference		1.00	Reference	
II	1.53	0.99	2.37	2.89	1.96	4.25
III	3.00	2.04	4.42	3.47	2.28	5.30
IV	4.88	3.73	6.39	21.48	10.75	42.93
CPR on arrival						
No	1.00	Reference		1.00	Reference	
Yes	4.88	3.47	6.87	11.81	3.69	37.86
Creatinine in µmol/L						
< 70	1.00	Reference		1.00	Reference	
70–105	0.76	0.53	1.10	0.72	0.44	1.19
106–140	1.81	1.24	2.64	1.39	0.82	2.38
141–176	2.46	1.55	3.92	1.22	0.62	2.41
177–353	2.84	1.73	4.68	1.25	0.59	2.64
≥ 354	3.92	2.09	7.34	4.58	2.53	8.31
Haemoglobin in g/dL						
< 10	1.00	Reference		1.00	Reference	
10–11	1.34	0.76	2.37	1.22	0.70	2.13
12–13	0.88	0.51	1.53	1.04	0.58	1.85
14–15	0.62	0.35	1.08	0.42	0.21	0.82
≥ 16	0.73	0.40	1.35	0.40	0.15	1.04

*CABG* coronary artery bypass grafting, *CI* confidence interval, *MI* myocardial infarction, *NSTEMI* non-ST-segment elevation myocardial infarction, *OR* odds ratio, *PCI* percutaneous coronary intervention, *STEMI* ST-segment elevation myocardial infarction

**Table 8:** Glucose-HbA1c ratio (GHR) as an independent predictor of 1-year all-cause mortality

	STEMI			NSTEMI		
	OR	95% CI		OR	95% CI	
GHR						
Below proposed cut-off	1.00	Reference		1.00	Reference	
Above proposed cut-off	2.28	1.80	2.89	1.30	0.92	1.83
Age in years						
< 40	1.00	Reference		1.00	Reference	
40–49	1.27	0.48	3.38	0.34	0.08	1.53
50–59	1.47	0.57	3.80	0.45	0.12	1.73
60–69	2.25	0.87	5.81	0.76	0.20	2.88
70–79	3.99	1.53	10.43	1.22	0.32	4.67
≥ 80	7.85	2.92	21.09	2.13	0.54	8.42
History of MI/PCI/CABG						
No	1.00	Reference		1.00	Reference	
Yes	1.19	0.90	1.59	1.53	1.12	2.10
Killip class on admission						
I	1.00	Reference		1.00	Reference	
II	1.49	0.96	2.31	2.86	1.95	4.21
III	2.46	1.66	3.66	3.22	2.09	4.93
IV	4.01	3.04	5.29	21.20	10.60	42.39
CPR on arrival						
No	1.00	Reference		1.00	Reference	
Yes	3.73	2.62	5.31	10.31	3.25	32.74
Creatinine in µmol/L						
< 70	1.00	Reference		1.00	Reference	
70–105	0.73	0.51	1.06	0.71	0.43	1.16
106–140	1.58	1.08	2.32	1.35	0.79	2.31
141–176	2.05	1.27	3.29	1.20	0.61	2.36
177–353	2.49	1.50	4.11	1.21	0.58	2.55
≥ 354	3.55	1.89	6.67	4.46	2.46	8.06
Haemoglobin in g/dL						
< 10	1.00	Reference		1.00	Reference	
10–11	1.21	0.68	2.14	1.27	0.72	2.21
12–13	0.81	0.47	1.41	1.10	0.61	1.96
14–15	0.56	0.32	0.99	0.44	0.22	0.88
≥ 16	0.67	0.37	1.24	0.42	0.16	1.10

*CABG* coronary artery bypass grafting, *CI* confidence interval, *GHR* glucose-HbA1c ratio, *MI* myocardial infarction, *NSTEMI* non-ST-segment elevation myocardial infarction, *OR* odds ratio, *PCI* percutaneous coronary intervention, *STEMI* ST-segment elevation myocardial infarction

**Table 9:** Stress-hyprglycaemia ratio (SHR) as an independent predictor of 1-year all-cause mortality

	STEMI			NSTEMI		
	OR	95% CI		OR	95% CI	
SHR						
Below proposed cut-off	1.00	Reference		1.00	Reference	
Above proposed cut-off	2.20	1.73	2.79	1.25	0.90	1.75
Age in years						
< 40	1.00	Reference		1.00	Reference	
40–49	1.24	0.47	3.31	0.34	0.08	1.52
50–59	1.45	0.56	3.74	0.44	0.11	1.71
60–69	2.21	0.86	5.69	0.75	0.20	2.84
70–79	3.88	1.49	10.15	1.22	0.32	4.63
≥ 80	7.79	2.90	20.91	2.11	0.54	8.32
History of MI/PCI/CABG						
No	1.00	Reference		1.00	Reference	
Yes	1.19	0.89	1.58	1.53	1.12	2.09
Killip class on admission						
I	1.00	Reference		1.00	Reference	
II	1.49	0.96	2.32	2.85	1.94	4.20
III	2.46	1.65	3.66	3.20	2.08	4.93
IV	4.06	3.08	5.36	21.26	10.64	42.50
CPR on arrival						
No	1.00	Reference		1.00	Reference	
Yes	3.72	2.62	5.30	10.29	3.24	32.71
Creatinine in µmol/L						
< 70	1.00	Reference		1.00	Reference	
70–105	0.72	0.50	1.05	0.71	0.43	1.16
106–140	1.57	1.07	2.31	1.35	0.79	2.30
141–176	2.07	1.29	3.33	1.20	0.61	2.36
177–353	2.44	1.47	4.03	1.21	0.58	2.55
≥ 354	3.51	1.86	6.59	4.43	2.45	8.01
Haemoglobin in g/dL						
< 10	1.00	Reference		1.00	Reference	
10–11	1.28	0.73	2.27	1.27	0.73	2.22
12–13	0.86	0.50	1.49	1.10	0.62	1.96
14–15	0.60	0.34	1.05	0.45	0.23	0.88
≥ 16	0.73	0.39	1.34	0.42	0.16	1.10

*CABG* coronary artery bypass grafting, *CI* confidence interval, *MI* myocardial infarction, *NSTEMI* non-ST-segment elevation myocardial infarction, *OR* odds ratio, *PCI* percutaneous coronary intervention, *SHR* stress-hyperglycaemia ratio, *STEMI* ST-segment elevation myocardial infarction

## Discussion

The main findings of our study were: glucose, GHR, and SHR were independent predictors of 1-year all-cause mortality in STEMI patients, whereas glucose and HbA1c were independent predictors in NSTEMI patients. AUC analysis showed GHR and SHR performed better than glucose in predicting 1-year all-cause mortality in diabetic STEMI patients, whereas glucose, GHR, and SHR performed equally well in non-diabetic STEMI patients. Furthermore, AUC analysis revealed that glucose performed better than HbA1c in predicting 1-year all-cause mortality in both diabetic and non-diabetic NSTEMI patients. The optimal values for glucose, GHR, and SHR in STEMI patients were 15.0 mmol/L, 2.11, and 1.68 in diabetic patients and 10.6 mmol/L, 1.72, and 1.51 in non-diabetic patients with a negative predictive value of > 94%. Similarly, the optimal values for glucose were 10.87 mmol/L and 8.1 mmol/L in diabetic and non-diabetic NSTEMI patients respectively.

It is postulated that hyperglycaemia leads to poorer outcomes after AMI events due to an acute increase in cortisol and catecholamine levels secondary to activation of the sympathetic nervous system as a physiological response to stress [23]. Catecholamines suppress insulin release from the pancreatic β-cells and promote hepatic and muscular glycogenolysis. This decreases glucose uptake into the heart and causes hyperglycaemia. Cortisol reduces glucose transporter translocation in the peripheral tissues and increases liver gluconeogenesis and hence hyperglycaemia [24–26]. It is speculated that the hyperglycaemia causes a poor outcome as it induces oxidative stress [27], increases endothelial dysfunction [28] and reduces the cardioprotective effect of ischaemic preconditioning [29].

### SHR and glucose as clinical outcome predictors

We found that the glucose and SHR were independent predictors of all-cause mortality at 1-year which corroborates the key finding from a recent report based on the Italian cohort where hyperglycaemia was an independent predictor of all-cause mortality in non-diabetic STEMI patients [30]. Therefore, glucose and SHR may improve the risk-stratification of STEMI patients. Clinicians can use this information to follow up on selected patients up more closely and be more aggressive in up-titrating goal-directed medical therapy and controlling their cardiovascular risk factors. The TIMI and GRACE risk scores have traditionally been used in prognosticating patients after acute myocardial infarction [31, 32]. Whether glucose or SHR adds independent prognostic value above the TIMI and GRACE risk scores needs to be validated in future studies.

Current guidelines provide a single glucose reading as a cut-off to define SH [2, 3]. There has also not been any clear protocol to date for the management of acute hyperglycaemia in AMI patients, with studies having conflicting results at best. Except for the DIGAMI-1 trial, results of subsequent larger randomized controlled trials with glucose-insulin-potassium infusions have been neutral or even caused increased hypoglycaemia rates [33–35]. One factor contributing to these results is that a single glucose cut-off was used. It may be worth considering recruiting patients based on the optimal glucose cut-off values or targeting the therapy to see if this would optimize outcomes in acutely hyperglycaemic patients. Our study support the findings by Hao et al. by demonstrating that there are different cut-off values for acute glucose in predicting adverse events at day 30 and year 3 depending on diabetic status [36]. Our study builds on the work by Hao et al. by studying 1-year all-cause mortality as a hard end-point, supporting the need for optimal glucose cut-off values separately for diabetic and non-diabetic STEMI patients.

Glucose and its related parameters are critical predictors for clinical outcomes following AMI. Hypoglycaemia on admission has been reported as a predictor of clinical outcomes in AMI patients [37–41]. Specifically, the prognostic value of hypoglycaemia was found mainly in diabetic patients on admission [38, 40, 41], and hypoglycaemia measured post-admission did not predict a worse outcome in AMI patients [39], indicating that hypoglycaemia may not be a direct mediator of the adverse outcomes and SHR may be a more reliable predictor for diabetic patients in general especially when only random glucose is available. In addition, the information on glycaemic status can be useful beyond prediction for short-term prognosis. Admission glucose is an independent predictor of long-term prognosis in non-diabetic AMI patients [42]. Interestingly, glucose variability between visits to clinic is associated with adverse cardiac remodeling after STEMI, suggesting that the clinical value of glucose and its related parameters is significant and should be investigated further [43].

Previously, Roberts et al. showed that SHR was an independent predictor of death or intensive care admission while glucose alone was not. A similar formula (random serum glucose/HbA1c) has subsequently been studied in a Korean post-AMI population of 4362 subjects from the COACT registry [44] and it showed that SHR predicted mortality, AMI and stroke in the non-diabetic STEMI population. An alternative method of calculating relative hyperglycaemia has been termed the glycaemic gap. This is calculated by subtracting the estimated average glucose levels over 3 months from the admission glucose [45]. Like the SHR, the glycaemic gap performs better than either admission glucose or HbA1c alone at predicting the risk of moderate-to-severe stroke [46]. Two recent publications have shown that using SHR as a biomarker showed increased mortality risks in diabetic Australian and Italian AMI patients [30, 47]. The former study was done on 192 patients in the HI-5 trial showing that relative but not absolute glycaemia during insulin treatment was associated with complications post-AMI [47]. The latter study consisted of 1553 consecutive AMI patients, and the study utilised a formula termed the acute-to-chronic glycemic ratio. Both studies showed that in AMI patients with diabetes, the glycemic ratio was a better predictor of in-hospital mortality than admission glycaemia [30]. Our study corroborates these findings that a metric adjusted for background glycemic control performs better in risk prediction. Further efforts are needed to standardize the use of a common metric of stress hyperglycaemia considering background glycemic control so that studies can be directly comparable and common definitions can be developed for future therapeutic studies.

### Strengths and limitations

The strength of this study is that the study cohort of AMI patients was derived from a national registry-level database, which allowed comprehensive and accurate case capture. The use of the national death registry to track death outcomes meant that there was no lost to follow-up. However, we acknowledge several limitations of this study. We could not exclude the possibility of selection bias given that more than half the patients in the database were excluded from the analysis due to missing data. As this was a retrospective study, causality cannot be determined in this study. We also could not standardize the time in which the acute glucose levels were measured in hospital within the first 24 h of presentation as we used retrospective data, although this does reflect real-world practice. Therefore, the data of AMI-induced hyperglycaemia should be interpreted in the context that the stress-induced hyperglycaemia lasts about 8 h and the in-hospital measurement was done within 24 h [48]. Moreover, the blood glucose was measured after the PCI procedure in a very small subset of the AMI patients. We did not have all the variables to compute the GRACE and TIMI risk scores in our cohort and therefore we could not assess whether glucose or SHR provided additive prognostic value over those existing scores. However, we did adjust for prognostic factors available from the SMIR cohort.

## Conclusion

In summary, in this national registry of AMI patients treated by PCI, glucose, GHR, and SHR were independent predictors of 1-year all-cause mortality in STEMI with acceptable negative and positive predictive values, whereas glucose was the only reliable predictor in NSTEMI patients. Our findings need to be validated in future studies.

## Supplementary Information

**Additional file 1: Figure S1.** Survival Curve for STEMI and NSTEMI Patients. The Kaplan–Meier curve was plotted for overall STEMI and NSTEMI patients (Panel A) or with their status of diabetes (Panel B). **Figure S2.** Plot of Hazard Ratio against Glucose. The hazard ratio for 1-year-all-cause mortality was plotted for STEMI and NSTEMI with their status of diabetes against their glucose level. **Figure S3.** Plot of Hazard Ratio against SHR. The hazard ratio for 1-year-all-cause mortality was plotted for STEMI and NSTEMI with their status of diabetes against their SHR. **Figure S4.** Plot of 1-year all-cause mortality against glucose, HbA1c, glucose-HbA1c ratio (GHR) and stress-hyperglycaemia ratio (SHR). The 1-year all-cause mortality was plotted for diabetic and non-diabetic patients against their glucose, HbA1c, GHR and SHR levels.

## Data Availability

The datasets used in this study are property of the National Registry of Diseases and were collected primarily for internal use. De-identified data can be accessed for public health research purposes after appropriate approval is obtained from the Institutional Review Board and Ministry of Health.
