# Supplementary material for: Optimal glucose, HbA1c, glucose-HbA1c ratio and stress-hyperglycaemia ratio cut-off values for predicting 1-year mortality in diabetic and non-diabetic acute myocardial infarction patients
Source: Cardiovasc Diabetol. 2021 Oct 19;20:211. doi: 10.1186/s12933-021-01395-3 (PMC8524932; doi:10.1186/s12933-021-01395-3)
Supplement: Supplementary file 1 — Additional file 1: Figure S1. Survival Curve for STEMI and NSTEMI Patients. The Kaplan–Meier curve was plotted for overall STEMI and NSTEMI patients (Panel A) or with their status of diabetes (Panel B). Figure S2. Plot of Hazard Ratio against Glucose. The hazard ratio for 1-year-all-cause mortality was plotted for STEMI and NSTEMI with their status of diabetes against their glucose level. Figure S3. Plot of Hazard Ratio against SHR. The hazard ratio for 1-year-all-cause mortality was plotted for STEMI and NSTEMI with their status of diabetes against their SHR. Figure S4. Plot of 1-year all-cause mortality against glucose, HbA1c, glucose-HbA1c ratio (GHR) and stress-hyperglycaemia ratio (SHR). The 1-year all-cause mortality was plotted for diabetic and non-diabetic patients against their glucose, HbA1c, GHR and SHR levels. [file 12933_2021_1395_MOESM1_ESM.docx]

**Figure S1**. **Survival Curve for STEMI and NSTEMI Patients**

**A**

**B**

Abbreviations: STEMI, ST-segment elevation myocardial infarction; NSTEMI, non-ST-segment elevation myocardial infarction

**Figure S2. Plot of Hazard Ratio against Glucose**

| All STEMI   | All NSTEMI   |
| --- | --- |
| Diabetic STEMI   | Diabetic NSTEMI   |
| Non-diabetic STEMI   | Non-diabetic NSTEMI   |

Abbreviations: STEMI, ST-segment elevation myocardial infarction; NSTEMI, non-ST-segment elevation myocardial infarction; SHR, stress-hyperglycaemia ratio

**Figure S3. Plot of Hazard Ratio against SHR**

| All STEMI   | All NSTEMI   |
| --- | --- |
| Diabetic STEMI   | Diabetic NSTEMI   |
| Non-diabetic STEMI   | Non-diabetic NSTEMI   |

Abbreviations: STEMI, ST-segment elevation myocardial infarction; NSTEMI, non-ST-segment elevation myocardial infarction; SHR, stress-hyperglycaemia ratio; stress hyperglycemia ratio, SHR

**Figure S4a. Plot of 1-year all-cause mortality against glucose by diabetic status**

**Figure S4b-i. Plot of 1-year all-cause mortality against Hba1c among diabetic patients**

**Figure S4b-ii. Plot of 1-year all-cause mortality against Hba1c among non-diabetic patients**

**Figure S4c. Plot of 1-year all-cause mortality against glucose-hba1c ratio (GHR) by diabetic status**

**Figure S4d. Plot of 1-year all-cause mortality against stress-hyperglycaemia ratio (SHR) by diabetic status**
